# Plant-produced RBD and cocktail-based vaccine candidates are highly effective against SARS-CoV-2, independently of its emerging variants

**DOI:** 10.3389/fpls.2023.1202570

**Published:** 2023-08-02

**Authors:** Tarlan Mamedov, Damla Yuksel, Irem Gurbuzaslan, Merve Ilgin, Burcu Gulec, Gulshan Mammadova, Aykut Ozdarendeli, Shaikh Terkis Islam Pavel, Hazel Yetiskin, Busra Kaplan, Muhammet Ali Uygut, Gulnara Hasanova

**Affiliations:** ^1^ Department of Agricultural Biotechnology, Akdeniz University, Antalya, Türkiye; ^2^ Institute of Molecular Biology and Biotechnologies, Ministry of Science and Education, Republic of Azerbaijan, Baku, Azerbaijan; ^3^ Department of Microbiology, Medical Faculty, Erciyes University, Kayseri, Türkiye; ^4^ Vaccine Research, Development and Application Center, Erciyes University, Kayseri, Türkiye

**Keywords:** SARS-CoV-2, plant produced RBD, antigen cocktail, virus neutralization, Delta, Omicron

## Abstract

Severe acute respiratory syndrome coronavirus 2 (SARS-CoV-2) is a novel and highly pathogenic coronavirus that caused an outbreak in Wuhan City, China, in 2019 and then spread rapidly throughout the world. Although several coronavirus disease 2019 (COVID-19) vaccines are currently available for mass immunization, they are less effective against emerging SARS-CoV-2 variants, especially the Omicron (B.1.1.529). Recently, we successfully produced receptor-binding domain (RBD) variants of the spike (S) protein of SARS-CoV-2 and an antigen cocktail in *Nicotiana benthamiana*, which are highly produced in plants and elicited high-titer antibodies with potent neutralizing activity against SARS-CoV-2. In this study, based on neutralization ability, we demonstrate that plant-produced RBD and cocktail-based vaccine candidates are highly effective against SARS-CoV-2, independently of its emerging variants. These data demonstrate that plant-produced RBD and cocktail-based proteins are the most promising vaccine candidates and may protect against Delta and Omicron-mediated COVID-19. This is the first report describing vaccines against SARS-CoV-2, which demonstrate significant activities against Delta and Omicron variants.

## 1 Introduction

The severe acute respiratory syndrome coronavirus 2 (SARS-CoV-2) that causes the coronavirus disease 2019 (COVID-19) infection, a highly infectious RNA virus, has undergone numerous mutations with the formation of genetically diverse linkages since first appearing in the city of Wuhan in 2019. Mutations could potentially have an impact on viral transmission as they affect the interaction between the S protein and its receptor, ACE-2. Among variants of concern (VOCs), the Alpha (B.1.1.7) and Delta (B.1.617.2) variants are associated with a high viral transmission rate and virulence compared to the parental Wuhan strain. Nine mutations were found in the S protein of the Delta variant, including in the amino-terminal domain (NTD) (five mutations) and receptor-binding domain (RBD) (two mutations, L452R, T478K). One mutation (P681R) was found near the furin cleavage site, and the other (D950N) in the S2 domain (Planas et al., 2021). Notably, mutations of G476, F486, T500, and N50, observed within the RBD are close to the receptor (ACE-2) binding site.

On 26 November 2021, a new variant named Omicron (B.1.1.529) was designated the fifth VOC (He et al., 2021), which carries more than 60 mutations compared to the original Wuhan strain. In the new Omicron variant, 36 mutations were found in the S protein sequence, including 30 amino acid substitutions, three deletions, and one insertion. It should be noted that 15 of the 30 amino acid substitutions are in the RBD (Tiecco et al., 2022).

Vaccination is currently the most effective way to prevent pathogenic diseases, including COVID-19. However, currently, available vaccines are less effective or not effective against emerging SARS-CoV-2 variants, such as the Delta strain (B.1.617) and the Omicron (B.1.1.529). Therefore, developing COVID-19 vaccines that are effective against emerging new variants of SARS-CoV-2, such as the Omicron, will be a very challenging task. Recently, we reported the successful production of RBD variants of the S protein of SARS-CoV-2 (Mamedov et al., 2021a) and of an antigen cocktail (Mamedov et al., 2021b) in *Nicotiana benthamiana*, which are highly produced in plants and elicited high-titer antibodies with potent neutralizing activity against SARS-CoV-2. Since the SARS-CoV-2 virus has mutated over time, in this study we tested how RBD or cocktail antigens could elicit specific immune responses against existing SARS-CoV-2 variants, Delta and Omicron. We demonstrate that plant-produced RBD or cocktail antigen-elicited antibodies are capable of neutralizing the Delta or Omicron variants.

## 2 Materials and method

### 2.1 Cloning, expression, and purification of gRBD, dRBD, and N+RBD proteins

Cloning, expression, and purification of glycosylated RBD (gRBD), deglycosylated RBD (dRBD), and N+RBD (nucleocapsid protein + receptor binding domain) proteins from *N. benthamiana* plant were performed as described recently (Mamedov et al., 2021a; Mamedov et al., 2021b). The genes encoding RBD of SARS-CoV-2 spike protein (RBD, 319-591 aa, GenBank: QHO60594.1) and nucleocapsid (N, 1–419 aa, GenBank: YP_009724397) were codon optimized using *N. benthamiana* codons and *de novo* synthesized (Biomatik Corp., Kitchener, ON, Canada). The signal peptide (MGFVLFSQLPSFLLVSTLLLFLVISHSCRA) of the tobacco *PR-1a* gene was added to the N-terminus of the RBD and N proteins. The KDEL sequence (ER retention signal) and the FLAG tag sequence (affinity purification tag) were added to the C-terminus. Genes were inserted into the plant expression pEAQ vector and introduced into the AGL1-1 strain of *Agrobacterium tumefaciens* to express genes in *N. benthamiana*. To produce the gRBD protein, pEAQ-RBD was infiltrated into *N. benthamiana* plant. To produce an N+RBD antigen cocktail, RBD and N genes were co-infiltrated into *N. benthamiana* leaves *via* co-agroinfiltration with both pEAQ-RBD and pEAQ-N constructs. To produce dRBD, the RBD and Endo H genes were co-infiltrated into *N. benthamiana* leaves *via* co-agroinfiltration with both pEAQ-RBD or pGreenII–Endo H constructs. Leaves were collected at 5 days after post-infiltration (dpi).

Expression levels of gRBD, dRBD, and N+RBD were determined by ELISA and Western blot analysis. To quantify the expression levels of gRBD, dRBD, and N+RBD, *N. benthamiana* plant leaf extract was filtered through Miracloth and then centrifuged at 20,000×*g* for 25 min at 4°C. The clear extract was analyzed by ELISA and Western blot.

For ELISA, plates were coated with 50 μl of diluted supernatants containing gRBD, dRBD, or N+RBD. The plates were also coated with commercially available RBD protein (Agr319-Phe541 aa, active protein, MBS2563882, MyBiosource, San Diego, CA, USA) as standard protein. Proteins were detected (i) using purified anti-FLAG antibody to detect plant-produced gRBD, dRBD, or N+RBD antigens; (ii) anti-SARS-CoV-2 S protein S1 mAb (cat. no. 945102, BioLegend, USA) to detect plant produced gRBD, dRBD antigens, and commercial RBD protein; (iii) or human novel coronavirus nucleoprotein (N) (1–419 aa) monoclonal antibody (MBS7135930, MyBioSource, San Diego, CA, USA) to detect plant-produced N protein.

For WB analysis, wells were loaded with 20 μl of diluted supernatants containing gRBD, dRBD, or N+RBD. The wells were also loaded with different amounts (100, 50, and 25 ng) of plant-produced, purified FLAG-tagged (Endo H or PNGase F) or commercially available RBD (Arg319–Phe541 aa, active protein, MBS2563882, MyBiosource) proteins as a standard protein. The expression levels of proteins were quantified using highly sensitive Gene Tools software (Syngene Bioimaging, UK). The expression levels were determined based on at least three replicates for each target protein.

Purification of plant-produced gRBD, dRBD, and N+RBD proteins was performed from 20 g of frozen leaves infiltrated with the pEAQ-RBD (with or without pGreenII-Endo H) or pEAQ-RBD+ pEAQ-N constructs, using anti-FLAG affinity chromatography as described recently (Mamedov et al., 2021a; Mamedov et al., 2021b). Plant-produced antigens were purified using anti-DYKDDDDK affinity gel (cat. no. 651503, BioLegend). gRBD-, dRBD-, and N+RBD-expressing leaves weighing 20 g were extracted in 1× PBS buffer and centrifuged at 13,000×*g* for 20 min at 4°C. The column was prepared and equilibrated with 10 column volumes of 1× PBS buffer. After washing the column with 10 CV of 1× PBS buffer, proteins from the column were eluted with 200 mM glycine buffer, pH 2.2, containing 150 mM NaCl. Eluted fractions were mixed with 2.0 M Tris for neutralization of glycine in elution buffer and concentrated. Purified antigens were analyzed on the SDS-PAGE and Western blotting.

### 2.2 SDS-PAGE and Western blot

Purified proteins were separated on 10% polyacrylamide gels. For SDS-PAGE analysis, gels were stained with Coomassie (Gel Code Blue, Pierce Rockford, IL, USA). For Western blot, separated samples were transferred to a polyvinylidene fluoride membrane and blocked with 1% I-block. The plant-produced antigens were detected with an anti-DYKDDDDK antibody (cat. no. 637301, BioLegend), followed by horseradish peroxidase (HRP)-conjugated anti-rat polyclonal antibody (cat. no. 405405, BioLegend). Proteins were visualized using the GeneGnome XRQ Chemiluminescence imaging system.

### 2.3 Immunogenicity studies

Immunogenicity studies of gRBD, dRBD, and N+RBD in mice were performed in groups of 6–7-week-old Balb/c male animals (six mice/group) as described recently (Mamedov et al., 2021a). Mice were immunized intramuscularly (IM) on days 0 and 21 with 5 μg of gRBD, dRBD, and N+RBD adsorbed to 0.3% Alhydrogel. Blood samples were taken from immunized mice on day 42 and used for microneutralization assay (MNT). Mice studies were conducted at Akdeniz University Experimental Animal Care in compliance with the ARRIVE guidelines and with the permission of the Animal Experiments Local Ethics Committee for Animal Experiments at Akdeniz (under protocol number 1155/2020.07.0) with the supervision of a veterinarian.

### 2.4 MNT assay

We used to live in the SARS-CoV-2 Wuhan (GB-MT327745; GISAID-EPI_ISL_424366), Delta (GB-OM945721; GISAID-EPI_ISL_10844545), and Omicron variants (GB-OM945722; GISAID-EPI_ISL_10844681). SARS-CoV-2 microneutralization (MN) tests were performed as previously described with minor modifications (Mamedov et al., 2021a). Before the day of the experiment, Vero E6 cells (ATCC, CRL-1586) (2×10^4^ cells/100 μl/well) were passaged to 96-well plates. The sera collected from vaccinated mice were heat-inactivated for 30 min at 56°C and subjected to twofold serial dilutions (from 1:4 to 1:1,024) with serum-free Dulbecco’s modified Eagle’s medium (DMEM). Twofold serial dilutions of the mice sera were mixed with an equal volume of DMEM containing 100 tissue culture infectious dose 50 (100 TCID50) of the SARS-CoV-2 variants and incubated for 90 min at 37°C. Following adsorption, inoculums were removed, and cells were incubated for 72 h with DMEM containing 2% FBS and checked for the cytopathic effect (CPE). Microplates were designed as follows: (i) to test two wells for each serum dilution, (ii) six control wells (virus only), and (iii) three wells of growth media for the blank. The CPE evaluation was performed according to the reduction of infection by 50% or more at the serum dilutions. The 50% microneutralization titer (MNT50) was analyzed by the Spearman–Karber method, which was calculated as the reciprocal of the highest serum dilution at which the infectivity was neutralized in 50% of the cell in wells.

### 2.5 Statistical analysis

We used GraphPad Prism software for statistical analysis. To compare the neutralization activity of gRBD-, dRBD-, and N+RBD-induced serums against live SARS-CoV-2 Wuhan, Delta, and Omicron variants, one way ANOVA test was used. Significant was accepted as *p* < 0.05, and *p*-values are shown as ^*^ *p* < 0.05; ^**^ *p* < 0.01; ^***^ *p* < 0.001. Each point on the graph was derived from three replicas for each dilution.

## 3 Results

**Figure 1:**
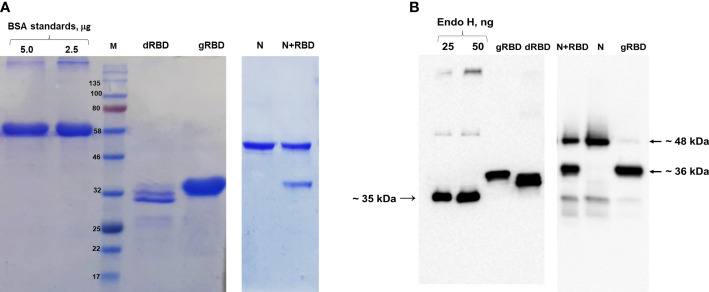
SDS-PAGE and Western blot analysis of gRBD, dRBD, and cocktail antigen (N+RBD) proteins, purified from *N. benthamiana* plant. Proteins were purified using anti-DYKDDDDK affinity gel. **(A)** SDS-PAGE for purified antigens. BSA was loaded as a standard, and target proteins were loaded as 5 μg. **(B)** Western blot analysis for plant-produced antigens. Endo H was used as a standard protein, and target proteins were loaded as 50 ng. Proteins were detected using the anti-FLAG antibody.

In this study, glycosylated and nonglycosylated versions of RBD and cocktail antigen obtained by co-expression of RBD with N protein were produced in *N. benthamiana*, as mentioned in our previous studies (**Figure 1**) (Mamedov et al., 2021a; Mamedov et al., 2021b).

**Figure 2:**
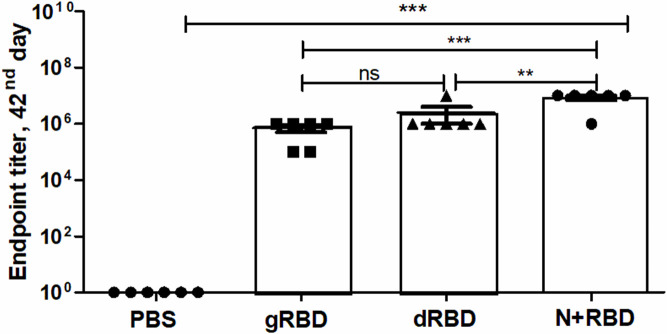
Immunogenicity of gRBD, dRBD, and N+RBD antigens in mice on the 42nd day. Mice were immunized with 5 µg of plant-produced proteins in a double injection on days 21 and 42. The endpoint titer was determined as the value corresponding to dilutions of the sera giving an OD value four times greater than the control mouse serum. Data in the graph are shown as the mean standard error of triplicates (SEM) at each sample dilution. The meaning of “**” is that the result is statistically significant. The meaning of “***” is that the result is highly statistically significant. ns, non significant.

The expression levels of gRBD, dRBD, and N+RBD, determined by ELISA and Western blot analysis, were ~42 mg/kg for dRBD and ~45 mg/kg for gRBD and cocktail antigens (N+RBD), similar to what was previously reported (Mamedov et al., 2021a; Mamedov et al., 2021b). RBD and cocktail antigens were purified from *N. benthamiana* by anti-FLAG affinity chromatography using anti-DYKDDDDK affinity gel, as described in Materials and methods. SDS-PAGE and Western blot analysis of the purified proteins are presented in **Figure 1** (and full length gels and blots in the **Supplementary Material**), which are consistent with the results we recently reported (Mamedov et al., 2021b). Immunogenicity studies of gRBD, dRBD, and N+RBD in mice were performed as described in Materials and methods and as recently reported (Mamedov et al., 2021a; Mamedov et al., 2021b). Antibody levels were determined by ELISA on 42nd-day mouse sera immunized with two doses of plant-produced RBD variants (gRBD or dRBD) or cocktail antigens (N+RBD). As demonstrated in **Figure 2**, the plant-produced gRBD, dRBD antigens, and cocktail antigens (N+RBD) were able to induce significantly high titers of antibodies with a 5-µg dose. As can be seen from **Figure 2**, the endpoint titer of N+RBD was 10^7^, higher than that of gRBD or dRBD. It should be noted that the endpoint titer of dRBD is higher than that of gRBD, which is consistent with the results we recently reported (Mamedov et al., 2021a).

**Figure 3:**
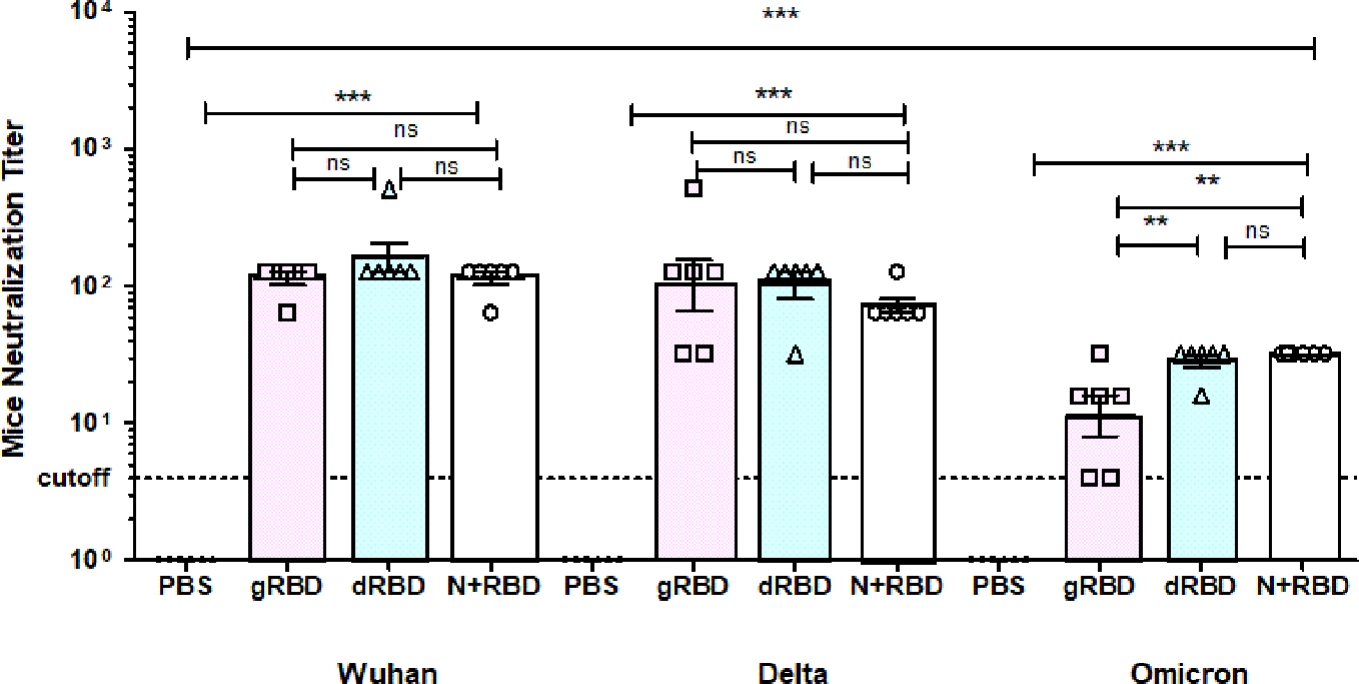
Sera from BALB/c mice immunized with gRBD, dRBD, and N+gRBD (cocktail) proteins demonstrated 50% microneutralization titers (MNT50) against live Wuhan, Delta, and Omicron variants of SARS-CoV-2. Microneutralization assay of 42nd-day mouse sera immunized with plant-produced gRBD, dRBD, and N+RBD (~2.7 μg + 2.3 μg, respectively) against live SARS-CoV-2 Wuhan, Delta, and Omicron variants as indicated. The experiment was performed using 4 to 1,024 dilutions of mouse sera collected on day 42. One-way ANOVA and Tukey’s multiple comparison tests were used to calculate statistical significance (*n* = 6 mice/group); ^***^ *p* < 0.001. The meaning of “**” is that the result is statistically significant.

To evaluate whether RBD or cocktail antigen-elicited antibodies are capable of neutralizing the Delta or Omicron variant, the latter being dominant at the moment, we tested Wuhan (GB-MT327745; GISAID-EPI_ISL_424366), Delta (GB-OM945721; GISAID-EPI_ISL_10844545), and Omicron variants (GB-OM945722; GISAID-EPI_ISL_10844681) with sera of mice immunized with two doses (5 μg per dose) of the plant-produced RBD variants or cocktail antigen-based COVID-19 vaccines (**Figure 2**). After two doses, Delta-neutralizing titers were not significantly reduced compared with Wuhan-neutralizing titers. Under the same conditions, Omicron-neutralizing titers were reduced by not more than fourfold compared with neutralizing titers in Wuhan (**Figure 3**). The plant-produced, deglycosylated RBD vaccine candidate was more effective compared to its glycosylated counterpart, suggesting the negative effect of *N*-glycosylation on protein functionality.

## 4 Discussion

Recently, we reported the successful production of glycosylated and deglycosylated RBD variants of the S protein of SARS-CoV-2 (Mamedov et al., 2021a) and an antigen cocktail (Mamedov et al., 2021b) in *Nicotiana benthamiana*, which are highly produced in plants and elicited high-titer antibodies with potent neutralizing activity against SARS-CoV-2. In this study, we demonstrate that plant-produced glycosylated and deglycosylated variants of RBD or cocktail antigen-elicited antibodies are capable of neutralizing the Delta or Omicron variant. It should be noted that after two doses (60 μg total synthetic mRNA), the Omicron-neutralizing titers of the messenger RNA (mRNA)-based COVID-19 vaccine (BNT162b2) were reduced by more than 22 times compared to Wuhan-neutralizing titers (Muik et al., 2022). Cele et al. (2022) demonstrated that in two-dose vaccinated individuals with the BNT162b2, the neutralization protection dropped over 40-fold against the Omicron versus the ancestral D614G virus. These results were expected given that the BNT162b2 vaccine is based on the full-length S protein sequence, while the S protein of the Omicron variant is highly mutated compared to other variants (Cele et al., 2022).

As we have described earlier, plant-produced dRBD demonstrated stronger binding to the SARS-CoV-2 receptor, ACE-2, compared with gRBD (Mamedov et al., 2021a). Moreover, we also demonstrated that both dRBD and dRBD variants induced strong neutralizing antibody responses in mice and sera immunized with the dRBD variant had higher neutralizing activity against live SARS-CoV-2 (Wuhan) compared with gRBD (Mamedov et al, 2021a). The impact of deglycosylation on neutralizing activity can be seen more significantly in the Omicron variant. When comparing Omicron-neutralizing titers, the plant-produced, deglycosylated RBD vaccine candidate was more effective compared to its deglycosylated counterpart, suggesting the negative effect of *N*-glycosylation on protein functionality. The negative effect of *N*-glycosylation on the functionality of proteins has been shown for several proteins. Deglycosylation, achieved by mutation of the *N*-glycosylation sites, increased the expression level and immunogenicity of the RBD protein vaccine (Zhang et al., 2020). The deglycosylation, achieved by the Endo H deglycosylation strategy (Mamedov et al., 2017) in plants significantly enhanced the efficacy of several proteins. For example, deglycosylation improved the SARS-CoV-2 neutralization activity of recombinant ACE2-Fc (Izadi et al., 2023). Deglycosylated Pfs48/45 of *Plasmodium falciparum*, produced in *N. benthamiana* plant using Endo H’s *in vivo* deglycosylation strategy had strong inhibition in SMFA, but under the same conditions, glycosylated Pfs48/45 did not display any significant inhibition (Mamedov et al., 2019b). Plant-produced glycosylated PA83 of *Bacillus anthracis* was not functionally active and was not able to combine with LF to form a lethal toxin (LeTx) and induce cell death (Mamedov et al., 2016). In contrast, *in vivo* PNGase F deglycosylated PA83 counterpart was functionally active and showed an EC_50_ value similar to the *Bacillus*-produced recombinant PA (Mamedov et al., 2016). When purified plant-produced PNGase (Mamedov et al., 2016) or Endo H (Mamedov et al., 2017) deglycosylated PA83 proteins were assessed for stability, they appeared to be more stable than the glycosylated counterpart. In addition, the plant-produced deglycosylated PA83 elicited significantly higher levels of TNA titers in immunized mice compared with its glycosylated counterpart (Mamedov et al., 2016). The negative effect of *N*-glycosylation was also demonstrated for plant-produced Pfs25 of *Plasmodium falciparum*. A nonglycosylated form of Pfs25 antigen (N-linked glycosylation sites mutated) generated higher antibody titers and enhanced TB activity compared to its glycosylated counterpart (Farrance et al., 2011). Likewise, the negative effect of *N*-glycosylation was also demonstrated for plant-derived human monoclonal antibodies directed against PA of *Bacillus anthracis*. Deglycosylated (N-linked glycosylation sites mutated), the plant-produced human monoclonal antibody demonstrated superior efficacy compared to a glycosylated form of this mAb in nonhuman primates (Mett et al., 2011).

Plant transient expression systems have proven to be promising alternative expression platforms for the expression of a wide variety of important recombinant proteins, including vaccines (Yusibov & Mamedov, 2010; Mamedov et al., 2016; Mamedov et al., 2019a; Mamedov et al., 2019b; Margolin et al., 2020), therapeutic proteins (Mamedov et al., 2019a), antibodies, and human and industrial enzymes. Importantly, plant expression systems have proven to be capable of high-level production of functionally active SARS-CoV-2 proteins (Mamedov et al., 2020; Mamedov et al., 2021a; Mamedov et al., 2021b) and ACE2 (Mamedov et al., 2021c), receptors of SARS-CoV-2 and SARS-CoV. We recently demonstrated the successful expression of glycosylated and deglycosylated forms of RBD and antigen cocktails, comprising RBD and nucleocapsid (N) proteins, as promising vaccine candidates against COVID-19. We demonstrated that RBD variants of the S protein of SARS-CoV-2 and cocktail antigens are highly produced in the *N. benthamiana* plant and elicited high-titer antibodies with potent neutralizing activity against SARS-CoV-2. Our hypothesis was that if any SARS-CoV-2 variant infects a human, this means that a mutation in the RBD region did not affect its binding to ACE2, the SARS-CoV-2 S protein receptor. Therefore, the correct selection of RBDs is critical for the successful production of functional RBDs with the ability to induce high neutralizing antibodies against SARS-CoV-2 and its variants. Since SARS-CoV-2 is an mRNA-based virus and mutations were expected, our strategy to eliminate possible emerging mutations was to select not the full sequence of the spike protein (most COVID-19 vaccine developers, including Pfizer-BioNTech and plant-based, Medicago’s VLP, which was approved for use by Health Canada, were targeted on the full-length S sequence) but rather a specific region of the S protein covering the RBD. Moreover, to address mutations, the N-protein + RBD multi-antigen, cocktail-antigen-based vaccine was developed and produced for the first time as a potential COVID-19 vaccine candidate (Mamedov et al., 2021b). In this study, we have shown that the N protein probably significantly contributes to the enhancement of neutralizing activity.

Since RBD of SARS-CoV-2 is the primary target for potent virus-neutralizing antibodies (Barnes et al., 2020; Brouwer et al., 2020; Piccoli et al., 2020; Wang et al., 2020; Yang et al., 2020), it is frequently used for COVID-19 vaccine development (Diego-Martin et al., 2020; Rattanapisit et al., 2020; Shin et al., 2021; Siriwattananon et al., 2021; Phoolcharoen et al., 2023) and as an antigen in serological assays and diagnostic reagents (Amanat and Krammer, 2020; Rattanapisit et al., 2020; Makatsa et al., 2021; Jirarojwattana et al., 2023). Several research groups have utilized *N. benthamiana* plants to produce different amino acid regions of RBD variants of SARS-CoV-2 (Diego-Martin et al., 2020; Rattanapisit et al., 2020; Shin et al., 2021; Siriwattananon et al., 2021). The amino acid sequence of R319-F541 is the most widely used sequence domain.

The S protein of SARS-CoV-2 contains a relatively high number of cysteine residues, and eight of the nine cysteine residues found in the RBD are involved in disulfide bridge formation (Lan et al., 2020). Notably, for some viruses, such as HIV, the redox state of the fusion protein was shown to be important for the viral fusion to the target cells (Ryser et al., 1994; Manček-Keber et al., 2021). Thus, the proper formation of disulfide bridges is critical for proper folding of the RBD. We produced the RBD variant (R319-S591 aa), where the number of cysteine residues is even, which forms correct disulfide bridges in the molecule that may stabilize the protein conformation, leading to a functional protein. In fact, as we recently reported, the expression levels of plant-produced gRBD and dRBD variants (Mamedov et al., 2020; Mamedov et al., 2021a) were higher than 45 mg/kg of fresh weight. The purification yields of plant-produced gRBD and dRBD were ~22 and ~20 mg pure protein/kg biomass, respectively, which demonstrate the commercialization feasibility of these vaccine candidates. Moreover, in mice, the plant-produced gRBD and dRBD antigens elicited high titers of antibodies with strong SARS-CoV-2 live virus-neutralizing activity (Mamedov et al., 2021a). We concluded that the correct choice of the amino acid sequence within the RBD of the spike protein is critical for achieving high-level production of soluble and functionally active protein (Mamedov et al., 2021a). On this point, in the study of Rattanapisit et al. (2020), RBD of the spike protein of SARS-CoV-2 was produced in *N. benthamiana* plant, and low yields (2–4 μg/g of fresh weight) were reported (Rattanapisit et al., 2020). In the mentioned study, the amino acid sequence (F318-C617) of RBD was not properly selected, and as a result, cytosine at position 617 remained unpaired (which should form a disulfide bond with cysteine residues at position 649 in the full-length S protein), which may destabilize the protein conformation and lead to a loss of functional activity. In fact, there was no report of neutralization of SARS-CoV-2 in this study. In another study, F318-C617 amino acids of RBD with the Fc region of human immunoglobulin G1 (IgG1) were selected for production in the *N. benthamiana* plant (Siriwattananon et al., 2021). As in the study of Rattanapisit et al., the amino acid sequence of RBD (F318-C617) was not properly selected, and as a result, cytosine at position 617 remained unpaired (Rattanapisit et al., 2020). The expression level of plant-produced SARS-CoV-2 RBD-Fc was low, at 25 μg/g of fresh weight. Low expression levels (2–4 mg/g of fresh weight) of RBD were also reported for RBD variant (His tagged, aa R319-F541, where the number of cysteine residues was not even) (Diego-Martin et al., 2020; Shin et al., 2021), which is too low to be economical for commercialization of these plant-produced RBD variants (Diego-Martin et al., 2020; Shin et al., 2021).

RBD, or full-length S1, of SARC-CoV-2-based VLPs have been also produced in *N. benthamiana* for COVID-19 vaccine development by different research groups (Royal et al., 2021; Moon et al., 2022; O'Kennedy et al., 2023). A number of plant-derived SARS-CoV-2 RBD-based antigens and VLPs are in preclinical and clinical trials (Gobeil et al., 2021; Ward et al., 2021; Hager et al., 2022; Pillet et al., 2022; Ruocco and Strasser, 2022). The studies showed that the plant-produced vaccine is safe without any severe allergic reactions and efficacious against SARS-CoV-2, including the Delta variant of concern (Gobeil et al., 2021; Ward et al., 2021; Hager et al., 2022; Pillet et al., 2022). The plant-produced VLP virus-like particle-based (two doses of 3.75 µg of antigen) vaccine has proven to show efficacy of 75.3% against COVID-19 (original Wuhan strain) and has been approved and authorized for use in Canada; however, there are no reports about the efficacy of this vaccine against Omicron. As this plant-based VLP vaccine is targeted on the full-length S sequence, Medicago is preparing to study an Omicron-adapted version of its vaccine, as reported by D’Aoust (https://www.reuters.com/business/healthcare-pharmaceuticals/canada-approves-medicagos-plant-based-covid-19-vaccine-adults-2022-02-24/) even though Canada has already approved a plant-based VLP Medicago vaccine (designed on S protein of SARS-CoV-2 original Wuhan strain) for COVID-19 for adults.

Collectively, all the above findings demonstrate that plant-produced RBD and cocktail antigens are cost-effective, safe, and promising vaccine candidates against SARS-CoV-2, independently of its variants, and may protect against Delta and Omicron-mediated COVID-19.

## 5 Conclusion

Since the SARS-CoV-2 virus has mutated over time, developing COVID-19 vaccines that are more effective against emerging variants is a very challenging task. Based on our previous and current studies, all our findings demonstrate that plant viral RBDs or cocktail antigens can be used as protein subunit vaccines to elicit specific immune responses against all existing SARS-CoV-2 variants, including Delta and Omicron.

## Data availability statement

The raw data supporting the conclusions of this article will be made available by the authors, without undue reservation.

## Ethics statement

Mice studies were conducted at Akdeniz University Experimental Animal Care in compliance with the ARRIVE guidelines, and with the permission of the Animal Experiments Local Ethics Committee for Animal Experiments at Akdeniz (under protocol number of 1155/2020.07.0) with the supervision of a veterinarian.

## Author contributions

TM conceptualized the study. TM designed the experiments. DY, MI, IG, BG, GM, AO, HY, BK, SP, and MU performed the experiments. TM and GH analyzed the data. TM and GH contributed to writing the paper. All authors contributed to the article and approved the submitted version.
