# Supplementary material for: Plant-produced RBD and cocktail-based vaccine candidates are highly effective against SARS-CoV-2, independently of its emerging variants
Source: Front Plant Sci. 2023 Aug 2;14:1202570. doi: 10.3389/fpls.2023.1202570 (PMC10433747; doi:10.3389/fpls.2023.1202570)
Supplement: Supplementary file 1 [file DataSheet_1.pdf]

Supplementary Material

Full-length gels and blots

1. Full-length gels

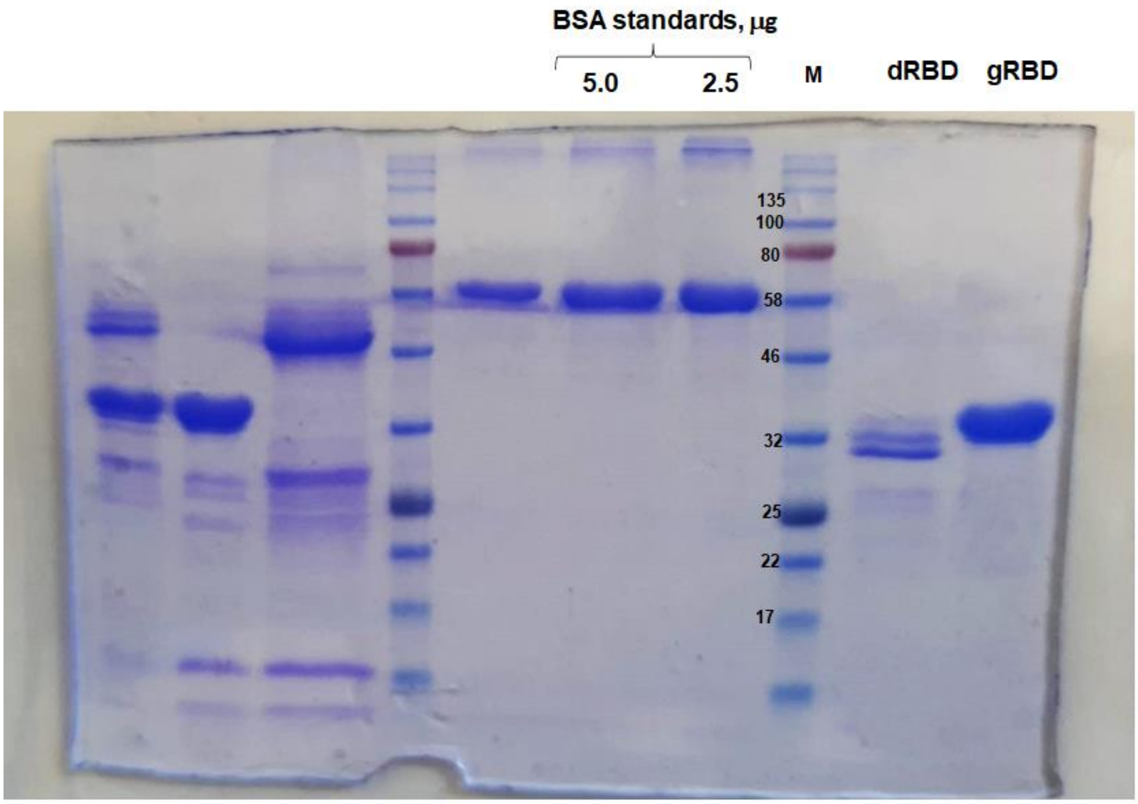

Supplementary Figure 1A. Figure 1A (left), indicated as BSA standards (2.5 and 5.0 μg) and dRBD, gRBD was cropped from this gel.

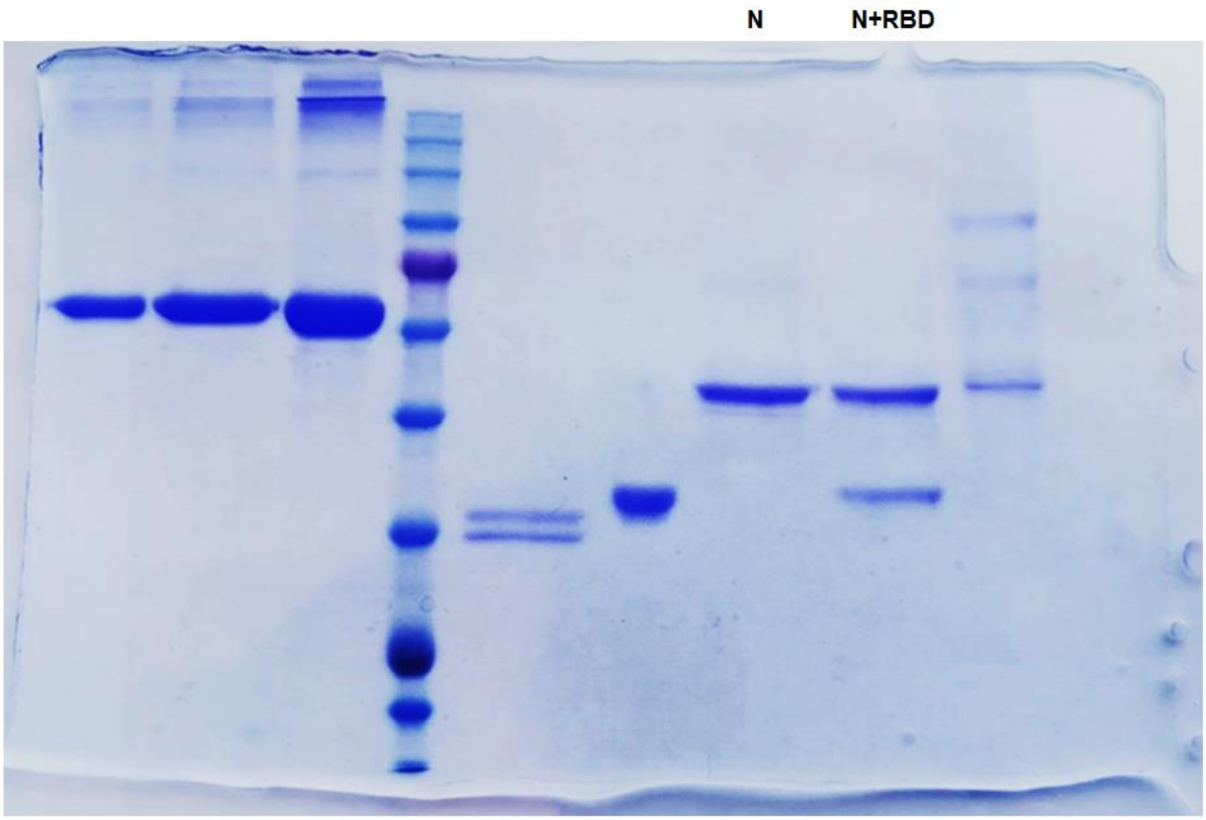

Supplementary Figure 1B. Figure 1A (right), indicated as N and N+RBD was cropped from this gel.

2. Full-length blots

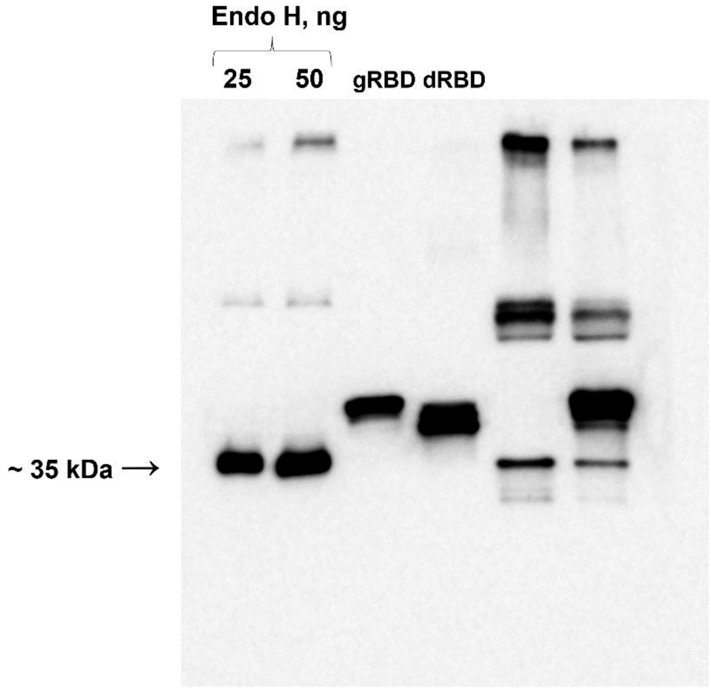

Supplementary Figure 2A. Figure 1B (left), indicated as Endo H (25 and 50 ng), gRBD and dRBD was cropped from this blot.

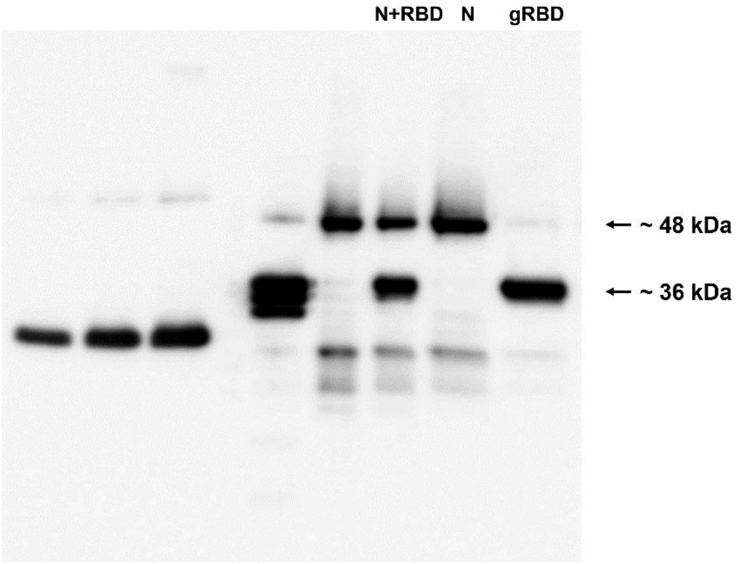

Supplementary Figure 2B. Figure 1B (right), indicated as N+RBD, N and RBD was cropped from this blot.
